# Assessing Malignant Risk in B3 Breast Lesions: Clinical Insights and Implications

**DOI:** 10.3390/jcm14010070

**Published:** 2024-12-26

**Authors:** Sabatino D’Archi, Beatrice Carnassale, Cristina Accetta, Paolo Belli, Flavia De Lauretis, Enrico Di Guglielmo, Alba Di Leone, Antonio Franco, Elisabetta Gambaro, Stefano Magno, Francesca Moschella, Maria Natale, Alejandro Martin Sanchez, Lorenzo Scardina, Marta Silenzi, Riccardo Masetti, Gianluca Franceschini

**Affiliations:** 1 Multidisciplinary Breast Centre, Dipartimento Scienze della Salute della Donna e del Bambino e di Sanità Pubblica, Fondazione Policlinico Universitario Agostino Gemelli IRCCS, 00168 Rome, Italy; 2 Dipartimento di Diagnostica per Immagini, Radioterapia Oncologica ed Ematologia, Fondazione Policlinico Universitario Agostino Gemelli IRCCS, 00168 Rome, Italy; 3 Dipartimento di Scienze Mediche e Chirurgiche, Università Cattolica del Sacro Cuore, 00168 Rome, Italy

**Keywords:** B3 breast lesions, diagnosis, imaging techniques, biopsy methods, breast surgery

## Abstract

**Background/Objectives**: B3 breast lesions, characterized by uncertain malignant potential, pose a significant challenge for clinicians. With the increasing use of preoperative biopsies, there is a need for careful management strategies, including watchful waiting, vacuum-assisted excision (VAE), and surgery. This study aims to assess the concordance between preoperative biopsy findings and postoperative histology, with a focus on evaluating the positive predictive value (PPV) for malignancy in B3 lesions. **Methods**: Over a seven-year period, 305 patients preoperatively diagnosed with B3 lesions were treated at the Multidisciplinary Breast Center of “Fondazione Policlinico Universitario Agostino Gemelli IRCCS” in Rome. All cases were reviewed at multidisciplinary meetings involving surgeons, radiologists, histopathologists, and oncologists. Preoperative diagnoses were obtained by ultrasound-guided core needle biopsies (CNBs) or stereotactic-guided vacuum-assisted biopsies (VABs). The radiological features were assessed using the Breast Imaging Reporting and Data System (BIRADS), and discrepancies between radiological and pathological findings were recorded. The biopsy results were compared with the postoperative histological findings to calculate the PPV for malignancy. **Results**: Of the 305 B3 lesions biopsied, 242 were confirmed as B3 on the final histological examination, resulting in a concordance rate of 79.3%. A total of 63 cases were upgraded to malignancy on postoperative histology, yielding a cumulative upgrade rate of 20.7%. The PPV for malignancy was 31.5% for atypical ductal hyperplasia (ADH), 27.6% for lobular neoplasia (LN), 22.9% for papillary lesions (PLs), 12.1% for flat epithelial atypia (FEA), 10.4% for radial scar (RS), and 10.3% for phyllodes tumors (PTs). **Conclusions**: Our findings demonstrate that the cumulative PPV for B3 lesions, as well as the PPV for each subtype, are consistent with the existing literature. The factors influencing the PPV include the use of CNB versus VAB, discordance between the BIRADS and biopsy results, the presence of atypia in the biopsy sample, the presence of microcalcifications on mammography, mass lesions identified on MRI, and the extent of the lesion. These factors should be considered in the personalized management of B3 lesions, potentially leading to more targeted and less invasive approaches in the future.

## 1. Introduction

B3 breast lesions refer to a group of abnormal findings identified on breast biopsy, characterized by heterogeneous histological and radiological features, uncertain malignant potential, and variable risk of progression to malignancy. These lesions are often identified through radiological imaging techniques such as microcalcifications or masses. They are usually diagnosed via preoperative biopsies performed under ultrasound or stereotactic guidance, which assist in informing subsequent treatment decisions. Common types of B3 lesions include atypical ductal hyperplasia (ADH), lobular neoplasia (LN) (classified by the WHO as atypical lobular hyperplasia (ALH) or classical lobular carcinoma in situ (LCIS), flat epithelial atypia (FEA), radial scar/complex sclerosing lesion (RS), papillary lesion (PL), and phyllodes tumors (PTs). In some cases, B3 lesions may coexist with malignant tumors or increase the risk of developing breast cancer (BC) with the likelihood of progression varying across different lesion types. Overall, the cumulative upgrade rate for B3 lesions ranges from 10% to 35% [1].

The rise in new diagnoses due to widespread screening programs, along with an increasing focus on treatment de-escalation, underscores the need for a clear approach to managing these lesions [2]. B3 lesions are particularly challenging due to the difficulty in obtaining a definitive histological diagnosis through core biopsy [3,4,5]. Studies have shown a notable underestimation of malignancy in B3 lesions; for example, El Sayed et al. reported a malignancy underestimation rate of 19.1% [6], while Bianchi et al. [7] observed a 21.2% positive predictive value (PPV) for B3 lesions diagnosed via CNB or VAB after surgical excision.

Over the past decade, significant changes have occurred in the management of B3 lesions. Previously, the uncertainty surrounding their malignant potential often led to surgical intervention. However, advances in radiological techniques now allow for less invasive options, including active surveillance in certain cases. Additionally, VAE has gained popularity, enabling the removal of a larger tissue sample and reducing the risk of diagnostic underestimation [1]. Given this progress, it is crucial for surgeons to be well-informed about alternative management strategies.

This study aims to assess the concordance between preoperative biopsy findings and postoperative histology, with a focus on evaluating the PPV for malignancy in B3 lesions in order to help guide clinical decisions.

## 2. Materials and Methods

We analyzed retrospectively 305 cases diagnosed with B3 on preoperative biopsy who underwent surgical excision at the Multidisciplinary Breast Center of the Fondazione Policlinico A. Gemelli IRCCS in Rome between January 2017 and December 2023. We defined B3 lesions following the worldwide, most commonly used pathologic classification for breast lesions, the B-classification. For each case, we collected the following data: age, familiarity, BIRADS, presence/absence of microcalcifications, preoperative MRI, size of the lesions, biopsy guidance, biopsy results, type of surgery performed, intraoperative histological examination, and postoperative histological results. All radiological images were acquired at the Multidisciplinary Breast Center of Policlinico Gemelli. Ultrasound guidance with a 14G needle was performed for 70.5% (215) of the biopsies. Stereotactic guidance VABB (29.5%, 90 patients) with an 8G needle was employed for lesions non-detectable by ultrasounds or microcalcifications. After every VABB for microcalcifications, a mammogram verified cores to find out whether the sample was representative. A post-procedural mammography shot was taken after every VABB to determine whether there was any residual lesion. The indications to perform surgery were established during a multidisciplinary meeting composed of oncologists, surgeons, radiotherapists, radiologists, pathologists, geneticists, and psychologists. In most cases, the decision for surgery was based on a BIRADS classification of 4 or 5, discrepancies between the imaging and histopathology results, residual lesions following VAB, an absence of prior evidence of the lesion in radiological examinations, and factors such as pain, discomfort, or patient preferences. We calculate the overall PPV and the PPV broken down for the B3 category with the following formula: (number of malignant cases) × 100/(total number of B3 cases).

## 3. Results

During the period between January 2017 and December 2023, 305 patients received a B3 diagnosis and a subsequent surgical procedure in our Breast Unit. Among the 215 CNBs and the 90 VABBs, the most frequent lesions were PL (83 diagnoses, 27.2%) and ADH (73 diagnoses, 23.9%), followed by RS (48, 15.7%), PT (18 benign and 21 border, in total 39, 12.8%), FEA (33, 10.8%), and LN (8 ALH and 21 classic CLIS, in total 29, 9.58%). The main characteristics of the included breast biopsies are presented in Table 1. The definitive histological evaluation confirmed the preoperative diagnosis in 242 cases (79.3%); however, 63 cases (20.7%) were found to be malignant. Malignant lesions were found in 23 ADH (36.5%), in 19 PLs (30.1%), in 8 LN (12.7%), in 5 RS (7.9%), in 4 FEA (6.4%) and in 4 PTs (6.4%). The surgical excision histological results for different B3 entities and the associated PPV for breast malignancy, including a comparison of the recent literature [3,4,8], are shown in Table 2. The final histological reports showed 32 cases of ductal carcinoma in situ (DCIS), 12 following an ADH diagnosis (37.5%), 13 following PL (40.6%), 4 following FEA (12.5%), two following RS (6.3%), and 1 following classic LCIS (3.1%). Seven definitive diagnoses of pleomorphic lobular carcinoma in situ (LCIS) were reported, most of them following LN evidence on biopsy (5–71.4%), with the remaining two diagnoses following in one case ADH (14.3%) and in the other RS (14.3%). The maximum radial extension of the in situ component was 40 mm. Invasive breast tumor was reported in 19 cases; in particular, only 2 were lobular subtypes (1 in ADH and 1 in LN). Invasive ductal carcinoma (IDC) has been predominantly found in ADH and PLs, respectively 9 (52.9%) and 6 (35.3%). In a single case, it was found in RS (5.9%) and the remaining case in LN (5.9%). The maximum radial extension of the invasive component was 24 mm. Upon a definitive histological examination, one case was compatible with squamous adenocarcinoma related to a preoperative biopsy of RS, and four cases resulted in a malignant phyllodes tumor. The characteristics of the upgraded lesions and predictive factors of malignancy are shown in Table 3. Based on our data, we estimated a cumulative upgrade rate of 20.7%. The estimated upgrade rate for each subtype of lesion at the preoperative biopsy was as follows, in decreasing order: 31.5% for ADH, 27.6% for LN, 22.9% for PL, 12.1% for FEA, 10.4% for RS, and 10.3 for PT. In the sample studied, reoperations for sentinel-node biopsy or reshavings of resection margins were necessary eight times. In the remaining cases, this was unnecessary, because we conducted an intraoperative histological analysis on the frozen section, revealing a malignant component. Conversely, in cases where patients underwent further surgery, the intraoperative evaluation did not reveal any malignant features. These findings highlight how intraoperative analyses can often prevent unnecessary additional interventions while promptly addressing any malignant findings.

## 4. Discussion

B3 lesions of the breast are composed of various pathological lesions characterized by an uncertain malignant potential, which may be linked to DCIS, pleomorphic LCIS, or invasive carcinoma (IC). The likelihood of being connected to malignant conditions varies significantly across different B3 lesion types, with an upgrade rate ranging from 10% to 35% [8]. These lesions have always represented a challenge for surgeons: in particular, in recent years, according to the increase of preoperative biopsies in daily practice, we are faced with the choice of whether to operate, perform VAE, or monitor over time. In the general perspective of de-escalation of surgery, the indications for surgery in B3 lesions have decreased, thanks to the introduction of percutaneous VAE, which allows for the complete removal of the lesion in cases of smaller lesions and spares patients from surgical intervention. For example, based on the 2018 NHS Breast Screening Multidisciplinary Working Group Guidance [9], most B3 lesions smaller than 20 mm should be managed with VAE. However, the indications are highly variable, and in most cases in clinical practice, the recommendation is for surgical removal. Numerous guidelines have been published about treating B3 lesions, which differ slightly from each other in indications. According to the latest European guidelines developed by EUSOMA, EUSOBI, ESSO, and ESP [10], the management of lesions diagnosed as B3 following preoperative biopsy varies depending on the presence of atypia, lesion size, sample size, patient preferences, and ranges from follow-up to VAE or surgery, to be discussed at a multidisciplinary meeting. Based on the Third Consensus Conference [4], if the CNB resulted in a B3 lesion, removal of the lesion was recommended by the panelists; in cases of ADH 100% (2018: 100%), FEA 92% (2018: 65%), LN 86% (2018: 67%), PL 92% (2018: 77%), PT 92% (2018: 98%), and RS 88% (2018: 60%), while the option of VAE is used as an alternative to surgery in routine clinical practice in selected cases. In this case series, we analyzed B3 lesions identified through preoperative biopsy, which were recommended for surgical intervention following a comprehensive multidisciplinary evaluation. This study aimed to assess the rate of upgrading observed in post-operative histological examinations and identify the associated predictive factors.

### 4.1. Atypical Ductal Hyperplasia (ADH)

ADH is one of the most frequently identified B3 lesions in breast pathology. It is often linked with clustered calcifications, masses, or irregular densities observed on mammograms. Histologically, it is characterized as a small, low-grade, clonal intraductal lesion, typically measuring 2 mm in maximum diameter or involving only segments of a terminal ductal–lobular unit. In our study, ADH represented 23.93% of all B3 preoperative diagnoses (73 lesions), and the cumulative upgrade rate was 31.5% (23 lesions), the highest in our cohort. Distinguishing the PPV based on the type of preoperative biopsy, we reported a PPV of 23.1% for VABB (9 lesions) and 24.3% for CNB (14 lesions), according to the evidence that CNB is widely acknowledged to entail a greater risk of underestimating the lesion grade in the final histological assessment. In the literature, the upgrade rate for ADH ranges from 18% to 87% for 14 G needles, contrasting with 10% to 39% for 11 or 9 G samples [11]. Essentially, it is unsurprising that providing a larger tissue sample decreases the chance of missing a diagnosis of DCIS or IC. Of all of the 23 ADH lesions that were later diagnosed as malignant tumors on the final histological examination, 11 (47.8%) were found to be associated with microcalcifications in the radiological preoperative evaluation, all of them with BIRADS-4 or BIRADS-5. Preoperative MRI was performed in 39 cases, particularly in 12 cases later diagnosed as B5 on the final histological examination. Among the MRI carried out in the B5 cases, 7 documented mass-like enhancement (6 BIRADS-4 and only 1 BIRADS-5), 3 documented non-mass-like enhancement (all of them BIRADS-4), and the remaining 2 were negative (BIRADS-1 or 2). In 10 of the 23 B5 cases (43.5%), there was radiological evidence of microcalcifications reported as BIRADS-4 or BIRADS-5. Among all malignant cases, 12 patients were in menopause (52.2%), and 6 (26.1%) had familiarity with breast malignancies. These results are concordant with the published literature. Numerous studies have documented that factors associated with an increased risk of upgrade in ADH include obtaining a smaller amount of biopsy tissue, mainly through CNB, persistence of calcifications after VAB, lesion size exceeding 15 mm on imaging, patient age over 50 years, and the presence of multifocality in the ADH biopsy specimen [12]. The carcinomas detected at the postoperative histological examination were 1 pleomorphic LCIS, 12 DCIS, 9 IDC, and 1 ILC with a median, radial extension of 34 mm (1 mm–55 mm); of these, 4 cases required reoperation for sentinel lymph node biopsy or margin re-excision. The remaining 19 cases did not require revision surgery due to the intraoperative evidence of malignancy on the frozen section histological examination.

### 4.2. Papillary Lesion (PL)

Papillary lesions (PLs) are typically observed in radiology as a well-defined nodule with or without cystic components or as a small mass within a dilated duct, histologically characterized by benign papillary architecture comprising fibrovascular cores covered by benign luminal epithelium with associated myoepithelium; they are typically classified as B3 due to their potential intralesional heterogeneity. The presence of associated epithelial atypia on biopsy is the most significant predictor for the upgrade to malignancy, which should be diligently sought and documented. When a papillary lesion lacks epithelial atypia, the likelihood of malignancy in the subsequent excision specimen is low, ranging from 9% to 13.2%. Conversely, when atypia is present, the upgrade rate substantially increases, ranging from 36% to 47.8% [2]. Recent series have reported a median upgrade rate for PLs without atypia to DCIS or IC of only 2.3%; in contrast, PLs with atypia exhibited a significantly higher upgrade rate to DCIS or IC, with a median of 26.9% [13,14]. In our study, PLs accounted for 27.2% of all B3 preoperative diagnoses (83 lesions), with an overall upgrade rate of 22.9% (19 lesions). On preoperative biopsies, 30 lesions were identified as PL with atypia, and 17 of these resulted in BC after surgery (PPV 56.7%). In comparison, 53 lesions were identified as PL without atypia on preoperative biopsy, and 2 resulted in BC after excision (PPV 3.8%). When differentiating the PPV based on the type of preoperative biopsy, we observed a PPV of 10% for VABB (one lesion) and 24.7% for CNB (18 lesions). In a recent meta-analysis, Zhang et al. [15] identified 10 predictive factors for an upgrade, including a BIRADS 4C or 5 category assignment, the presence of a mass or calcifications on mammography, bloody nipple discharge, radio-pathological discordance, peripheral lesion location, palpable mass, and lesion size exceeding 1 cm. The upgrade rates associated with these predictive factors ranged from 7.3% to 31.1%. In our cohort, out of the 19 PLs subsequently identified as malignant tumors on the final histological examination, 2 (10.5%) were associated with microcalcifications in the radiological preoperative evaluation, all classified as BIRADS-4. Preoperative MRI was performed in a total of 33 cases, particularly in only 5 cases that were later diagnosed as B5 on the final histological examination, and all of them showed mass-like enhancement (1 classified as BIRADS 3, 1 as BIRADS 5, and the remaining 3 as BIRADS 4). Of the B5 cases, 9 patients were menopausal (47.4%), and 6 (31.6%) had a family history of breast malignancies. The carcinomas identified at the postoperative histological examination included 13 DCIS and 6 IDC: none of these cases required additional surgery, because an intraoperative histological examination was performed, diagnosing invasiveness. One case did not undergo reoperation because the patient was 80 years old, and there was no evidence of suspicious lymph nodes in the axillary ultrasound evaluation performed once the definitive histological result was obtained. Another case did not undergo reoperation due to the small size of the tumor (0.3 mm).

### 4.3. Radial Scar (RS)

RS typically manifests on mammography as a stellate lesion or region of architectural distortion, occasionally accompanied by calcifications. These imaging features warrant caution, as RS can mimic invasive BC. Histologically, central fibroelastosis is surrounded by compressed glandular structures and cysts, occasionally linked with sclerosing adenosis, benign epithelial hyperplasia, atypia, or malignant alterations [16]. The upgrade rate of RS heavily relies on the presence of associated atypical epithelial proliferation. The reported data on the upgrade rate from RS to DCIS or invasive cancer vary widely, ranging from 0% to 40% [17]. Numerous studies have shown that RS upgrade rates increase when atypia is present. Racka et al. [16] reported a 9% malignant outcome following surgical excision of RS without atypia on CNB, while those with atypia had an upgrade rate of 36%. According to the NHSBSP Guidelines, upgrade rates in cases of atypia were 36% compared to 10% without atypia [9]. Similar results were found in other studies, showing upgrade rates of 28% for RS with atypia versus 4% without atypia [18], as well as in a recent study on a large patient cohort, observing upgrade rates of 9% in RS without atypia versus 33% in RS with atypia [19]. In our research, RS constituted 15.7% of all B3 preoperative diagnoses (48 lesions), with a combined upgrade rate of 10.4% (5 lesions). On preoperative biopsy, 12 lesions were identified as RS with atypia, and 3 of these resulted in BC after surgery (PPV 25%). In comparison, 36 lesions were identified as RS without atypia, and only 2 resulted in BC after excision (PPV 5.6%). When analyzing the PPV based on biopsy type, we observed a PPV of 28.6% for VABB (2 lesions) and 7.3% for CB (3 lesions). Out of the 5 RS lesions later diagnosed as malignant tumors on the final histological examination, 3 (60%) were associated with microcalcifications in the radiological preoperative evaluation, all categorized as BIRADS-4 or BIRADS-5. Preoperative MRI was conducted in a total of 34 cases, particularly in 4 cases later diagnosed as B5 on the final histological examination. Two MRIs showed suspicious BIRADS 4 mass-like areas, and the remaining two cases correlated with BIRADS-4 microcalcifications on mammography and did not reveal any pathological or suspicious enhancement. Of the B5 cases, 1 patient was postmenopausal (20%), and 4 (80%) had a family history of breast malignancies. The carcinomas detected in the postoperative histological examination included 1 pleomorphic CLIS, 2 DCIS, 1 IDC, and 1 adenosquamous carcinoma with a median, radial extension of 24 mm (5 mm–45 mm); among these, one case required reoperation for sentinel lymph node evaluation and margin excision reshaving. The remaining four cases did not necessitate revision surgery due to the intraoperative confirmation of malignancy via frozen section histological examination.

### 4.4. Flat Epithelial Atypia (FEA)

FEA is commonly observed with other dubious lesions and shares imaging characteristics with both malignant and benign lesions. Histologically, FEA is categorized within the columnar cell lesion spectrum of the breast, which encompasses columnar cell alteration and hyperplasia, devoid of atypia; however, the presence of atypia designates it as FEA [20]. The conversion rate for FEA remains somewhat ambiguous. FEA is frequently linked with ADH, LN, and low-grade DCIS, and the likelihood of progression is closely tied to concurrent proliferative lesions [21]. At the same time, the risk of advancing to carcinoma is minimal for pure FEA. Wahab et al. [22] conducted the most extensive meta-analysis, including 42 studies with 2482 cases, revealing an upgrade rate of 5% for pure FEA on CNB after surgical excision. In contrast, Verschuur-Maes et al. [23] reported a 17% upgrade rate in a systematic review. Other recent review articles and meta-analyses have indicated that the upgrade rate following surgical excision ranged from 1% to 16% [24,25]. In our investigation, FEA comprised 10.8% of all B3 preoperative diagnoses (33 lesions), with a cumulative upgrade rate of 12.1% (4 lesions). When differentiating the PPV based on biopsy type, we documented a PPV of 14.3% for VABB (2 lesions) and 10.5% for CNB (2 lesions). Like other B3 lesions, FEA diagnosed on CNB requires further sampling, because detecting coexisting proliferative lesions elevates the upgrade rate. Of the 4 FEA lesions later diagnosed as malignant tumors on the final histological examination, 2 (50%) were associated with microcalcifications in the radiological preoperative evaluation, all classified as BIRADS-4. Preoperative MRI was conducted in 15 cases, particularly in 2 cases later diagnosed as B5 on the final histological examination: one showed suspicious mass-like enhancement (BIRADS 4), and the other did not show any pathological capitation. Of the B5 cases, none of the patients were in menopause, and 2 (50%) had a family history of breast malignancies. The carcinomas detected at the postoperative histological examination were all DCIS, with a median, radial extension of 17 mm (3 mm–26 mm); further surgical intervention was necessary in none of the cases.

### 4.5. Lobular Neoplasia (LN)

LN is typically mammographically undetectable and is frequently an incidental finding on biopsies. It generally appears as a non-palpable, invisible lesion, sometimes associated with microcalcifications on mammograms. It is a B3 lesion, considered a non-obligate precursor to BC, and based on this extent, the WHO categorizes it into ALH and LCIS. Non-classical LN lesions (pleomorphic, apocrine, or florid LCIS) are potential differential diagnoses classified as B5a lesions, with distinct management and a higher rate of progression [20]. LN lesions serve as risk factors, conferring an 8-10-fold increase in relative risk compared to the general population [26]. The upgrade rate after an LN diagnosis on breast biopsy varies widely in the literature, ranging from 0 to 50% [27]. Higher upgrade rates, ranging from 13 to 18%, are observed for LN associated with mass lesions or calcifications [28]. The most significant indicator of an upgrade to invasive cancer is a radiological discrepancy, such as a suspicious mass on imaging with a histopathologic diagnosis of LN obtained via CNB. In our investigation, LN represented 9.5% of all B3 preoperative diagnoses, encompassing 29 lesions (8 ALH and 21 LCIS), with a combined upgrade rate of 27.6% (8 lesions). When categorized by biopsy type, the PPV was 30% (6 lesions) for VABB and 22.2% (2 lesions) for CNB. Of the 8 lesions later identified as malignant in the final histological examination, 7 (87.5%) were associated with microcalcifications observed in the preoperative radiological evaluation, all classified as BIRADS 3-4. MRI was performed in 11 cases based on BIRADS suspicion, lesion size, or breast complexity. All cases classified as B5 in the final histological evaluation had a preoperative MRI evaluation (3 with non-mass-like and 2 with mass-like enhancement, all categorized as BIRADS-4). Of the B5 cases, half of the patients were postmenopausal, and 4 (50%) had a family history of BC. The final histological results showed that 5 malignant lesions were pleomorphic LCIS, 1 DCIS, and 1 IDC requiring reoperation for sentinel node biopsy. One lesion was LCIS but did not require further surgery due to the presence of an invasive component identified during the intraoperative histological evaluation. The median radial extension of the malignant lesions was 20 mm (ranging from 2 mm to 30 mm).

### 4.6. Phyllodes Tumors (PTs)

A PT radiologically presents as a well-defined, round, or oval mass without calcifications. Based on attributes such as margins, mitotic activity, stromal cellularity, and the ratio of epithelial to stromal components, the WHO (20) categorizes PTs into benign, typically in differential diagnosis with fibroadenomas, borderline, and malignant. While malignant PTs are classified as B5 lesions, benign and borderline are considered B3. The upgrade rate to malignancy after diagnosing benign PTs on CNB or VAB is uncommon. In the literature, the management of these lesions had primarily considered the “upgrade rate”, which refers to the proportion of phyllodes tumors detected in the final histological examination when a CNB yielded an equivocal result where “a phyllodes tumor could not be excluded”. There is considerable variability in the literature. After a final histological examination, Rakha et al. demonstrated that 37% of fibroepithelial lesions on CNB were identified as phyllodes tumors [2]. Still, only one lesion out of 52 was malignant. In our study, phyllodes tumors (PT) accounted for 12.8% of all B3 preoperative diagnoses (39 lesions), with a cumulative upgrade rate of 10.3% (4 lesions). On CNB, 18 lesions were identified as benign PTs, and just 1 resulted in malignant PT after surgery (PPV 5.6%). In comparison, 21 lesions were identified as borderline PTs on CNB, and 3 resulted as malignant PTs after excision (PPV 14.3%). All patients underwent CNBs. Out of the 4 PT lesions that were ultimately identified as malignant through the final histological examination, only one had undergone an MRI, which revealed a suspicious mass with contrast enhancement. Of the B5 cases, one patient was postmenopausal, and none had a family history of BC. All B5 lesions found in the postoperative histological examination were malignant PTs, with a median size of 150 mm. None of the cases required reoperation.

## 5. Conclusions

Our study highlights the need for a thoughtful and tailored approach to managing B3 breast lesions. These lesions are complex with varying malignant potential across different subtypes. Understanding the lesion-specific positive predictive values helps clinicians better assess the risks associated with each subtype. When deciding on the best management approach, key factors such as biopsy method, radiological discrepancies, and lesion characteristics (like atypia and microcalcifications) must be carefully considered. These insights suggest that personalized risk-based strategies can offer less invasive and more targeted treatments for patients with B3 lesions [29]. Looking ahead, the integration of advanced diagnostic tools and refined management protocols could lead to more precise patient-centered care, reducing unnecessary procedures and improving outcomes. As our experience shows, the future of managing B3 lesions may involve balancing active surveillance with targeted intervention guided by thorough multidisciplinary evaluations. This evolving field challenges clinicians to stay updated on new techniques and to consider all diagnostic information when determining the most appropriate treatment for each patient.

## Figures and Tables

**Table 1:** Characteristics of 305 breast biopsies.

Characteristics	N (%)
Radiological findings	
Masses	226 (74.1%)
Microcalcification	79 (25.9%)
BIRADS	
3	83 (27.2%)
4	211 (69.2%)
5	11 (3.6%)
Biopsy technique	
VAB	90 (29.5%)
CN	215 (70.5%)
Age at diagnoses (median)	48
Familiarity	
Y	88 (28.9%)
N	217 (71.1%)
Fertile age	
Y	188 (61.6%)
N	117 (38.4%)

**Table 2:** Surgical excision histological results for different B3 entities and associated PPV for breast malignancy, including a comparison of the recent literature.

	B3 Subtype	Cases with Surgical	Upgraded Cases and PPV for
		Excision	Malignancy (%)
Elsarkawy et al., 2020 [3]	ADH	15	6 (40%)
	LN	6	2 (33%)
	FEA	9	2 (22%)
	RS	4	0
	PL	38	6 (15%)
	PT	3	0
	Total	75	16 (21%)
Lucioni et al., 2021 [4]	ADH	32	13 (41%)
	LN	14	4 (21%)
	FEA	3	2 (66%)
	RS	1	0
	PL	2	0
	PT	1	0
	Total	68	19 (28%)
Bellini et al., 2023 [8]	ADH	259	103 (39.8%)
	LN	222	44 (19.9%)
	FEA	146	17 (11.6%)
	RS	145	7 (4.8%)
	PL	124	30 (24.4%)
	PT	53	7 (13.2%)
	Total	966	210 (22.7%)
This study	ADH	73	23 (31.5%)
	LN	29	8 (27.6%)
	FEA	33	4 (12.1%)
	RS	48	5 (10.4%)
	PL	83	19 (22.9%)
	PT	39	4 (10.3%)
	Total	305	63 (20.7%)

**Table 3:** Characteristics of lesions upgraded to B5 and predictive factors of malignancies.

B3 Subtypes Upgraded to B5	BIRADS 4-5	CNB	VAB	Atypia on Biopsy	MRI Mass Area	MRI Non-Mass Area	MRI Not Performed	Microcalcification	cT1a	cT1b	cT1c	cT2
23 ADH	23/23 (100%)	14/23 (60.9%)	9/23 (39.1%)	23/23 (100%)	7/23 (30.5%)	3/23 (13%)	13/23 (56.5%)	11/23 (48.7%)	0/23 (0%)	11/23 (47.8%)	6/23 (26.1%)	6/23 (26.1%)
8 LN	6/8 (75%)	2/8 (25%)	6/8 (75%)	0/8 (0%)	2/8 (25%)	2/8 (25%)	4/8 (50%)	7/8 (87.5%)	0/8 (0%)	2/8 (25%)	5/8 (62.5%)	1/8 (12.5%)
4 FEA	4/4 (100%)	2/4 (50%)	2/4 (50%)	0/4 (0%)	0/4 (0%)	1/4 (25%)	3/4 (25%)	2/4 (50%)	0/4 (0%)	2/4 (50%)	0/4 (0%)	2/4 (50%)
5 RS	5/5 (100%)	3/5 (60%)	2/5 (40%)	3/5 (60%)	2/5 (40%)	0/5 (0%)	3/5 (60%)	3/5 (60%)	0/5 (0%)	1/5 (20%)	3/5 (60%)	1/5 (20%)
19 PL	12/19 (63.2%)	18/19 (94.7%)	1/19 (5.3%)	10/19 (52.6%)	4/19 (21.1%)	0/19 (0%)	15/19 (78.9%)	2/19 (10.5%)	1/19 (5.3%)	6/19 (31.6%)	10/19 (52.5%)	2/19 (10.6%)
4 PT	4/4 (100%)	4/4 (100%)	0/4 (0%)	0/4 (0%)	1/4 (25%)	0/4 (0%)	3/4 (75%)	0/4 (0%)	0/4 (0%)	0/4 (0%)	2/4 (50%)	2/4 (50%)
63 Total	54/63 (85.7%)	43/63 (68.2%)	20/63 (31.7%)	36/63 (57.1%)	16/63 (25.4%)	6/63 (9.5%)	41/63 (65.1%)	25/63 (39.7%)	1/63 (1.6%)	22/63 (35%)	26/63 (41.2%)	14/63 (22.2%)

## Data Availability

The data presented in this study are available on request from the corresponding author.
